# Palmitic acid decreases cell migration by increasing RGS2 expression and decreasing SERCA expression

**DOI:** 10.1590/1678-4685-GMB-2020-0279

**Published:** 2021-03-15

**Authors:** Octavio Galindo-Hernandez, Ana Gabriela Leija-Montoya, Tatiana Romero-Garcia, Jose Gustavo Vazquez-Jimenez

**Affiliations:** 1 Autonomous University of Baja California, Laboratory of Biochemistry, School of Medicine, Campus Mexicali, BC, Mexico.; 2 Autonomous University of Baja California, Laboratory of Biochemistry, Sports School, Campus Mexicali, BC, Mexico.; 3 Autonomous University of Baja California, Laboratory of Molecular Pathogenesis, School of Medicine, Campus Mexicali, BC, Mexico.

**Keywords:** Palmitic acid, cell migration, RGS2, SERCA

## Abstract

Palmitic acid, the main saturated fatty acid, is related with a wide range of metabolic disorders such as obesity, type 2 diabetes and heart disease. It is known that palmitic acid disturbs the expression of some important proteins for cell homeostasis such as SERCA and RGS2, however, the role of this lipid at the molecular level in these disorders is not completely elucidated. Thus, our aim was to determinate the effect of palmitic acid in a relevant cell process as it is cell migration and the participation of SERCA and RGS2 in this response. We found that palmitic acid reduces cell migration (determined by the Boyden chamber method) in an epithelial cell line (HEK293) and this effect is modulated by SERCA and RGS2 differential protein expression (measured by western blot). Also, overexpression of individual proteins, RGS2 and SERCA, produced a decrease and an increase on cell migration, respectively. Taken together, these data suggest that the expression of regulatory proteins is affected by high concentrations of saturated fatty acids and in consequence cell migration is diminished in epithelial cells.

Obesity is a state that gives rise to the development of many metabolic conditions; such as hypertension, heart disease and type 2 diabetes mellitus (de Luca and Olefsky 2008; Ryu *et al*., 2019), however, the molecular alterations that precede these pathologies are not completely understood. Recently, it has been shown that a high-fat diet induces an increase in the expression of the Regulator of G protein signaling 2 (RGS2) (Nunn *et al*., 2011). RGS-proteins are GTPases accelerating proteins for heterotrimeric -subunits (Soundararajan *et al*., 2008). RGS2 selectively inhibits signaling mediated by heterotrimeric G_q/11_ proteins (Heximer *et al*., 1997).

There is a correlation between high concentrations of fatty acids (FA) and the elevation of RGS2 protein levels since the expression of this protein was increased in high-fat fed mice. Likewise, an RGS2 knock-out mouse model (*rgs2* ^*-/-*^ ) showed a reduction in serum lipids and increased insulin sensitivity (Nunn *et al*., 2011). Therefore, changes in RGS2 expression correlate with the pathogenesis of metabolic disorders (Osei-Owusu *et al*., 2007).

In this same context, obesity prompts augmented plasma levels of non-esterified FA, particularly palmitic acid (PA), the main saturated FA. Recent studies have shown that increased PA levels result in a reduced expression of the sarco/endoplasmic reticulum calcium ATPase (SERCA) and hence a decrease in its activity (Li *et al*., 2004; Fu *et al*., 2012; Mayer and Belsham 2010; Vazquez *et al*., 2016). Among other conditions, the endoplasmic reticulum (ER) requires a high luminal Ca^2+^ concentration to guarantee the correct synthesis, folding and assembly of membrane and secretory proteins (Ceylan-Isik *et al*., 2011). In this respect, SERCA protein plays a central role, since it is in charge of Ca^2+^ transport into the ER. Disturbances in ER homeostasis leads to stress and therefore activation of the unfolded protein response (UPR) (Özcan *et al*., 2004; 2006; Park *et al*., 2010), which directly induces ER stress, through a mechanism that has been proposed to be associated with the downregulation of the insulin signaling pathway (Vazquez *et al*., 2016).

Given that, we have focused on the study of RGS2 and SERCA proteins, considering that high concentrations of saturated fatty acids have recently been reported to be involved in the regulation of the expression of these proteins (Nunn *et al*., 2011; Vazquez *et al*., 2016). Nevertheless, there are no studies that integrate these two alterations and that attribute any immediate functional effect to them. In order to solve this, we used an epithelial cell line and PA as a study model; since the epithelia are in constant migration and regeneration, and these are the first to suffer alterations induced by PA (Duan *et al*., 2017; Ghezzal *et al*., 2020). Specifically, we have chosen embryonic kidney cells 293 (HEK293), because it is a cell line that possesses morphological and functional properties of epithelial cells (Braun and Huber, 2002; Stepanenko and Dmitrenko 2015) and also due to their efficiency to evaluate the expression of RGS2 and SERCA (Jang *et al*., 2014; Bovo *et al*., 2020). Furthermore, this cell line is convenient both for carrying out transfection and for evaluating cell migration (Xue *et al*., 2019; Wang *et al*., 2020).

Palmitic acid (≥99% pure), bovine serum albumin-fatty acid-free (FAF-BSA), Dulbecco's modified Eagle's medium (DMEM; Sigma Aldrich); fetal bovine serum (FBS; ByProductos); Lipofectamine 2000 (Life Technologies); full-length human SERCA2b cDNA clone (ID 5503508) in pCMV-SPORT6 vector (Invitrogen); full-length human RGS2 3xHA-tagged cDNA (CloneID RGS020TN00) in the pcDNA3.1+ vector (cDNA Resource Center); anti-SERCA2 (Thermo Scientific), anti-RGS2, anti-Actin and mitomycin C (Santa Cruz Biotechnology).

HEK293 cells (ATCC) were grown in P-100 dishes at 37°C in a humidified atmosphere (95% air, 5% CO_2_) in DMEM 10% FBS supplemented, 100 μg/ml streptomycin and 100 units/ml penicillin. For experiments, cells were sub-cultured in 6 plates to 80% confluence and cultured with DMEM (serum free) for 6 h and then PA treatments were performed.

HEK293 cells were seeded (2.5×10^4^) for 3 days. Then, cells were transfected with RGS2 3xHA tagged or pEF1/His-A- hSERCA2b cDNA (1 μg/well) in 5 μg/ml of Lipofectamine 2000 in complete culture medium at 37 °C for 6 h. This procedure was followed by 18 h culturing in fresh medium before lipid incubation.

PA stock solutions at 500 mM were prepared in DMSO. HEK293 cells were swapped to serum-free DMEM with 1% FAF-BSA and then treated with 0.25 mM of PA for 8 h. Then the cells were placed on ice, washed twice with ice-cold PBS and lysed with 100 μl of 1x Laemmli sample buffer.

Cell lysates were defrosted, sonicated, then incubated at 99 °C for 5 min, and finally centrifuged at 14,000 rpm for 5 min. The obtained supernatant was loaded on SDS-PAGE gels, electrophoresed and then transferred to PVDF membranes using a semi-dry chamber. Membranes were incubated with the corresponding primary antibodies overnight at 4 °C and washed 3 times with TBS-T buffer before incubating with secondary antibodies (HRP-conjugated) for 1 h at room temperature. Chemiluminescent signals were visualized using an HRP chemiluminescent western blot detection (Millipore). Densitometric signals of immunoblot films were determined with ImageJ Software (1.53e version).

Migration assays were performed by the Boyden chamber method in 24-well plates containing 12 cell culture inserts (Corning Inc., Cat. 3422). After starvation, HEK293 cultures were treated for 2 h with 12 μM mitomycin C to inhibit proliferation. Briefly, HEK293 cells (1x10^5^) were placed on the top chamber (100 μl/insert), whereas the lower chamber contained 600 μl DMEM supplemented or not with 10% FBS. Cells were incubated for 24 h at 37 °C with 5% CO_2_; and then cells on the upper surface of membrane were discarded, while cells on the lower surface of membrane were washed and fixed with cold methanol. The cells were stained with 0.1% crystal violet in 1X PBS, and the dye was eluted with 10% acetic acid. Finally, the solution was analyzed by a spectrophotometer at 600 nm. Background value was determined from wells with no attached cells.

PRISM 6.0 software was used to analyze the average absorbances as well as the densitometric intensities from western blot films. Statistical significance was determined by one-way ANOVA with Dunnett's post-test. Significance was defined at *p* value <0.05. Data were normalized based on the control and the mean ±S.E.M.

In this research we have explored two previously reported molecular events: First, the association between obesity and increased expression of RGS2 (Imagawa *et al*., 1999; Nishizuka et al., 2001; Nunn *et al*., 2011). Second, the association among high concentrations of PA and decreased SERCA2 expression (Vazquez *et al*., 2016).

We wanted to resolve whether these two molecular alterations were present in HEK293 cell line and in that case if they are associated with disturbances on cell migration. Thus, we performed incubations of HEK293 cells with 0.25 mM of PA for 8 h. Previously, this concentration was reported to cause functional modifications and protein expression alterations; however, this effect did not occur with high concentrations of unsaturated fatty acid (Vazquez *et al*., 2016). Our results showed that incubations with PA decrease HEK293 cells migration (Figure 1A). Also, as shown in Figure 1B, PA increased RGS2 expression ~3-fold over control; in the same experiment, the fatty acid decreased SERCA2 expression ~0.7-fold with respect to control (Figure 1C). These data suggest that PA decreases cell migration while increasing RGS2 and decreasing SERCA expression in HEK293 cells.

**Figure 1.**
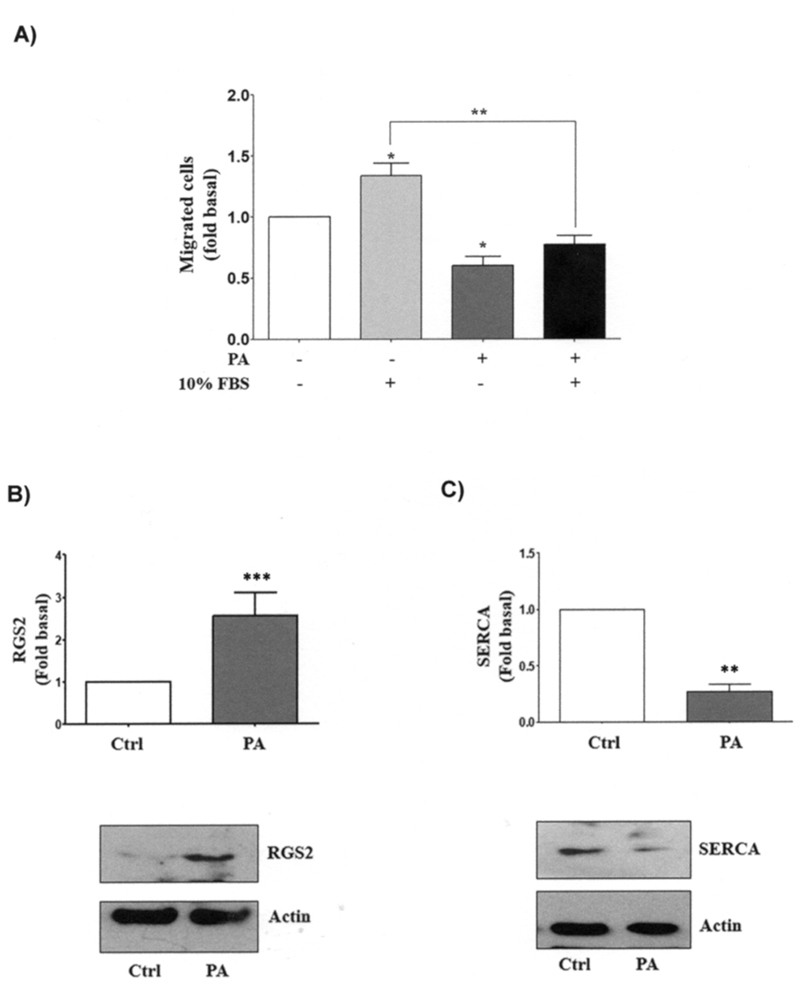
Palmitic acid inhibits cell migration in HEK293 cells. **a**. After starvation, HEK293 cells were washed, equilibrated in DMEM without FBS, and pretreated for 2 h with 12 μM mitomycin C. Cells were untreated or treated with 0.25 mM PA (an inducer of cell migration) or 10% FBS and cell migration assays were performed by using the Boyden chamber method. The graphs represent the mean ± S.E.M. of migration of three independent experiments and are expressed as the fold of unstimulated cells. **b**, **c** After starvation, HEK293 cells were washed and equilibrated in DMEM without FBS. Cells were treated with 0.25 mM PA in DMEM and lysates were obtained. RGS2 and SERCA were analyzed by western blotting with anti-RGS2 and anti-SERCA2, respectively. Western blots were also probed for actin as a loading control. Asterisks denote comparisons made to unstimulated cells. *p<0.05, **p<0.01, ***p<0.001 vs control condition (unstimulated cells).

Recent research has shown that PA induces a decrease in SERCA protein levels; as well as PA produced a sustained inhibition of SERCA pump ATPase activity (Vazquez *et al*., 2016).

On the other hand, it has been suggested that RGS2 may play an essential role in the regulation of body metabolism, as *rgs2* ^*-/-*^ mice metabolic activity was increased (Nunn *et al*., 2011).

To resolve whether alterations in the expression of SERCA and RGS2 were mechanisms associated with alterations in cell migration, transfection of both proteins in HEK293 cells was carried out. As shown in Figure 2A, transfection was efficient, nonetheless, the SERCA intensity signal was so strong that the control signal was only observed after overexposure of the plate. As shown in Figure 2B, in presence of 10% FBS, SERCA overexpression increases cell migration ~1-fold over control. On the other hand, overexpression of RGS2 decreased cell migration (~0.5-fold with respect to the control) (Figure 2B). Importantly, in 10% FBS conditions, overexpression of RGS2 decreases migration by ~1.5-fold respecting the SERCA overexpression condition (Figure 2B). It should be noted that these changes in migration were not present when overexpression of the empty vector was performed (MOCK) (Figure 2B). These data suggest that increased SERCA expression enhances cell migration, while RGS2 increased expression diminish it.

**Figure 2.**
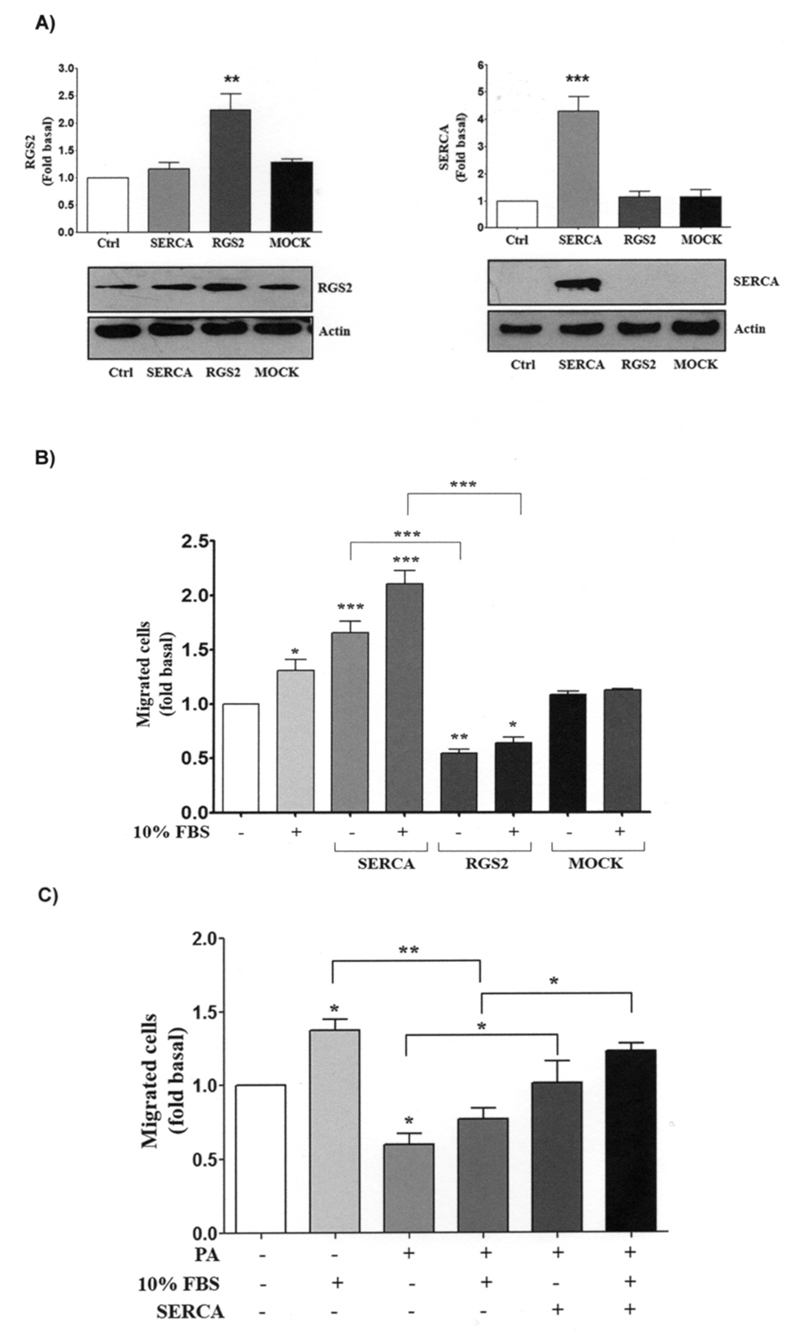
SERCA overexpression prevented the decrease in PA-induced cell migration in HEK293 cells. HEK293 cells were transfected with a RGS2 and SERCA2 construct or with an empty vector (MOCK). **a** Cells were lysed and RGS2 and SERCA expression were analyzed by western blotting using anti-RGS2 and anti-SERCA2 Ab, respectively. Western blots were also probed for actin as a loading control. **b** HEK293 cells were untreated or treated with 10 % FBS (as a positive control of cell migration) and cell migration assays were performed by using the Boyden chamber method. The graphs represent the mean ± S.E.M. of migration of three independent experiments and are expressed as the fold of unstimulated cells. **c** HEK293 cells were transfected with a SERCA2 construct prior to incubation for 8 h with 0.25 mM PA. Cell migration assays were performed by using the Boyden chamber method. The graphs represent the mean ± S.E.M. of migration of three independent experiments and are expressed as the fold of control. *p<0.05, **p<0.01, ***p<0.001 vs control condition (unstimulated cells).

Previous research has reported that SERCA overexpression prevents PA-induced insulin resistance (Park *et al*., 2010; Vazquez *et al*., 2016) and since PA decreases cell migration, we wanted to resolve whether SERCA overexpression prevents PA-induced decreased cell migration. Hence, we performed SERCA overexpression prior to incubation with PA. As shown in Figure 2C, SERCA overexpression prevented the decrease in cell migration induced by PA.

In conclusion, high concentrations of saturated fatty acids alter the expression of regulatory proteins, thus decreasing cell migration in epithelial cells.
